# Determinants of Knowledge, Attitude, and Practices of Veterinary Drug Dispensers toward Antimicrobial Use and Resistance in Main Cities of Malawi: A Concern on Antibiotic Stewardship

**DOI:** 10.3390/antibiotics12010149

**Published:** 2023-01-11

**Authors:** Henson Kainga, Marvin Collen Phonera, Ibrahim Chikowe, Elisha Chatanga, Hlupikire Nyirongo, Mike Luwe, James Mponela, Vincent Kachisi, Nathani Kamanga, Julius Chulu, Gilson Njunga, Daisy Nabadda, Alyce Fonchin, Simegnew Adugna Kallu, Steward Mudenda, Rabecca Tembo, Mildred Zulu, Florence Mwaba, Natalia Mbewe, Prudence Mpundu, Mulemba Tillika Samutela, Musso Munyeme, John Bwalya Muma, Edgar Simulundu

**Affiliations:** 1 Department of Veterinary Epidemiology and Public Health, Faculty of Veterinary Medicine, Lilongwe University of Agriculture and Natural Resources, Lilongwe 207203, Malawi; 2 Department of Animal Health and Livestock Development, Ministry of Agriculture, Lilongwe 207203, Malawi; 3 Pharmacy Department, Kamuzu University of Health Sciences (KUHES), Blantyre P.O. Box 360, Malawi; 4 Department of Veterinary Pathobiology, Faculty of Veterinary Medicine, Lilongwe University of Agriculture and Natural Resources, Lilongwe 207203, Malawi; 5 Excel Private Secondary School, Blantyre 30758, Malawi; 6 Department of Biosecurity, Ecosystems and Public Health, College of Veterinary Medicine, Makerere University, Kampala P.O. Box 7062, Uganda; 7 Community Initiative for Sustainable Development, Manko-Bamenda P.O. Box 356, Cameroon; 8 College of Veterinary Medicine, Haramaya University, Dire Dawa P.O. Box 138, Ethiopia; 9 Department of Pharmacy, School of Health Sciences, University of Zambia, Lusaka P.O. Box 50110, Zambia; 10 Department of Pathology and Microbiology, School of Medicine, University of Zambia, Lusaka P.O. Box 50110, Zambia; 11 Department of Basic and Clinical Nursing Sciences, School of Nursing Sciences, University of Zambia, Lusaka P.O. Box 50110, Zambia; 12 Department of Environmental and Occupational Health, Levy Mwanawasa Medical University, Lusaka P.O. Box 33991, Zambia; 13 Department of Biomedical Sciences, School of Health Sciences, University of Zambia, Lusaka P.O. Box 10101, Zambia; 14 Department of Disease Control, School of Veterinary Medicine, University of Zambia, Lusaka P.O. Box 10101, Zambia; 15 Macha Research Trust, Choma P.O. Box 20100, Zambia

**Keywords:** agrovet shops, antibacterials, antimicrobial resistance, antimicrobial stewardship, attitudes, knowledge, Malawi, practices

## Abstract

Antimicrobial resistance (AMR) is an emerging challenge to global public health. The use of antibiotics in the veterinary field is one of the contributing factors to AMR mostly due to poor knowledge, attitudes, and practices (KAP) of dispensers. Veterinary drug dispensers are expected to guide clients on indications, contraindications, and withdrawal periods of veterinary drugs. This study assessed veterinary drug dispensers’ KAP toward AMR and associated potential contributing factors. A cross-sectional study, using a structured questionnaire, was conducted in three main cities of Malawi, namely Mzuzu, Lilongwe, and Blantyre. A total of 68 agrovet shops were selected using a simple random sampling technique. The KAP level was presented descriptively. Bivariate and multivariable analyses were run to investigate the relationships between the independent and outcome variable. Overall, the KAP score for knowledge, attitude, and practices was 46.7%, 49.2%, and 41.6%, respectively. The significant determinants of the knowledge were the practice of asking for a written prescription (OR: 16.291, 95% CI: 11.6–24.2) (*p* = 0.024), female (OR: 0.609, 95% CI: 0.3–0.9) (*p* = 0.001), and old age (≥35) (OR: 0.227, 95% CI: 0.1–0.5) (*p* = 0.04). Poor knowledge, negative attitude, and poor practices were observed among most of the participants. Sensitization and training on AMR and antimicrobial stewardship are recommended to address the KAP score gaps and the observed determinants among veterinary drug dispensers.

## 1. Introduction

Antimicrobial resistance (AMR), an emerging public health challenge is increasing worldwide [1,2,3,4]. Globally, it was estimated to cause 4.95 million human deaths in 2019 [5]. Furthermore, it is projected that by 2030, AMR could force up to 24 million people into extreme poverty [6] and is likely to kill 10 million people per year by 2050 [2,3]. Bacterial AMR (antibiotic resistance) affects the globe negatively and accounts for appreciable challenges in treating human and livestock infections, food insecurity, and the weakened healthcare system [7,8]. Antibiotics (one type of antimicrobials) are experiencing resistance in all fields of life (humans and animals), leading to increased morbidity and mortality worldwide [5,9]. Of special concern is the reported resistance to many of the first-line antibiotics for treating bacteremia and other life-threatening infections [5].

In the wake of AMR burdens, several sub-Saharan African countries developed AMR preparedness plans, and East African countries reported the strongest AMR response [10,11]. In contrast, southern African countries had a weak AMR response [10,11]. To mitigate this, it is proposed that the AMR control strategy should be collectively implemented by member countries of the Southern African Development Community (SADC) [12]. One major challenge to tackling AMR is the lack of understanding of the true burden of this problem, particularly in locations where surveillance is minimal and data are sparse. The situation is more challenging mostly in countries that have weak or no AMR surveillance in animals and the environment [13,14].

Malawi has the most comprehensive data sets for AMR burden in sub-Saharan Africa, which depicts the growing trend of AMR burden in humans [15,16,17,18]. The AMR trends have shown a recent rise in third-generation cephalosporin (3GC) resistance among enterobacteria from 2003 to 2016. Musicha et al. [19] reported an increasing trend of resistance for the 3GC from 0.7% to 30.3% for *Escherichia coli* and from 11.8% to 90.5% for *Klebsiella* species. Kumwenda et al. [20] also reported that the prevalence of resistant *Klebsiella* species was (17.4%, *n* = 393), *Staphylococcus aureus* (34.7%, *n* = 783), *Proteus* species (11.4%, *n* = 256), and *Streptococcus pneumoniae* (60.3%, *n* = 44). Furthermore, Musicha et al. [19] reported that most bacteria exhibited high resistance to all regularly used antimicrobials excluding ciprofloxacin. In addition, despite having a paucity of information on AMR burden in animals, the rise in demand for food of animal origin has given impetus for increased livestock production [20,21], which has subsequently led to an increase in demand for antimicrobial use for improving animal health and increased production. In Malawi, livestock farmers value their livestock so much that they use available medicines to maximize production and remain solvent in a precarious economic and social environment [21,22,23].

Malawi has unclear AMR surveillance in animals and the environment, despite having less stringent regulations on accessing veterinary drugs [20,22,23]. Veterinary drugs are jointly regulated by the Pharmacy and Medicine Regulatory Authority (PMRA) and the Department of Animal Health and Livestock Development (DAHLD). The two institutions regulate the use of antimicrobials by ensuring that farmers buy veterinary drugs upon getting a prescription from a veterinary officer [20,22,23]. Regrettably, this is not the case, as most of the farmers access veterinary drugs without written prescriptions or advice from veterinary officers [22,23]. These practices may lead to drug misuse or abuse, which is likely to produce “superbugs”, contrary to the objectives of Malawi’s national antimicrobial strategy [22,23,24,25]. Veterinary drug dispenser shops dominantly operate as general veterinary drug shops that stock a variety of drugs, possibly targeting all species of animals. In addition, few specialized veterinary drug shops meet requirements for specific species such as poultry. These two groups differ in their mode of operation but complement each other to satisfy the demands of animal owners.

The prudent use of veterinary drugs depends on the knowledge of the veterinary drug dispensers as well as on the awareness and knowledge of the livestock farmers [26,27]. Most of the farmers interact with shop attendants when buying veterinary drugs, such that farmers trust the shop attendants’ drug recommendations and drug administration procedures [26]. These interactions are believed to be key to the prevention and controlling misuse of antimicrobials; hence, they contribute to combating AMR. The veterinary drug dispensers may be key to AMR monitoring and successful antimicrobial stewardship. Understanding the underlying determinants of AMR knowledge, attitude, and practices (KAP) among veterinary drug dispensers may guide the development of context-specific interventions to prevent and control AMR-promoting practices [26]. However, it is not clear whether the veterinary drug dispensers are familiar with AMR and their KAP toward AMR remains speculative. It is against this background that this study was formulated to assess the KAP toward AMR among the veterinary drug dispensers in the main cities of Malawi.

## 2. Results

### 2.1. Sociodemographic Characteristics of Study Participants from Agrovet Shops

The study recruited 68 respondents from agrovet shops. The geographical distribution of the participants included 24 from Blantyre, 32 from Lilongwe, and 12 from Mzuzu. Of the participants, 30.9% (21/68) were female and 69.1% (47/68) were males. The general agrovet shops were the majority 94.1% (64/68) compared to poultry drugs only and feed shops 5.9% (4/68). More than half of agrovet shops 82.4% (56/68) were located within the town centers, while 17.6% (12/68) were located within the residential areas (Table 1).

### 2.2. Commonly Dispensed Antibiotics

**Figure 1:**
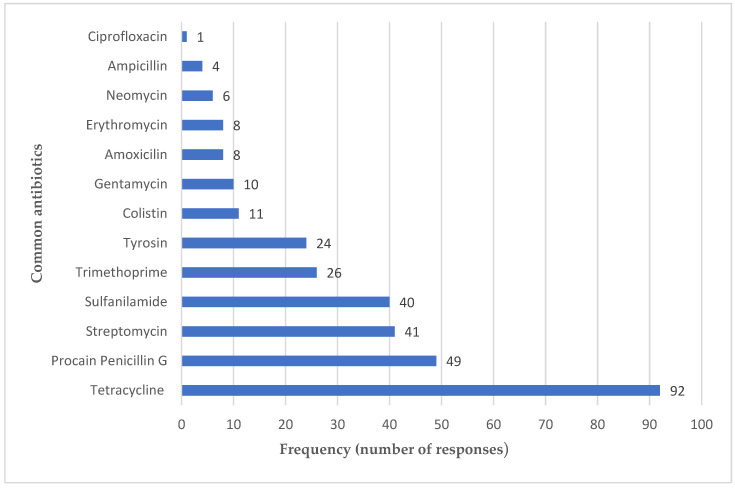
Distribution of commonly dispensed antibiotics by agrovet shops.

The overall frequency (calculated based on the number of responses from question 1 of section C of the questionnaire, Supplementary Material File S1) of common antibiotics was 331 and was observed in retail agrovet shops for animal health and livestock production purposes. The most dispensed class of antibiotics were tetracycline at 27.8% (92/331), followed by procaine penicillin G (PPG) at 14.8% (49/331), with ciprofloxacin being the least dispensed at 0.3% (1/331) (Figure 1).

Furthermore, the study assessed the number of brands of antibiotics commercially available in the agrovet shops (calculated based on the number of product names regardless of whether the product contained the same active ingredient and not on sample size). The study found a higher frequency of oxytetracycline brands 27.9% (12/43) among the available antibiotic brands. The concentrations of oxytetracycline among the brand names varied a lot which included 2%, 5%, 8%, 10%, 12.5%, and 20%. Other antibiotic brands were sulfa-333 20.9% (9/43), penstrep 9.3% (4/43), gentaject 6.9% (3/43), and colistin 4.6% (2/43), and among least was aliseryl 2.3% (1/43). Aliseryl was observed in all poultry-drugs-only veterinary product shops that participated in the study.

### 2.3. Participants’ Knowledge of Antimicrobial Use and Resistance

The majority of the participants 75.0% (51/68) were aware of the uses of antibiotics, and 48.5% (33/68) knew that antibiotics could not cure all diseases that affect the livestock. Lilongwe city had a higher KAP score of 90.6% (29/32) compared to the other two cities for knowledge of the curative effects of antimicrobials (*p* = 0.037). About three-quarters of participants, 76.5% (52/68), knew about AMR and its occurrence in livestock and humans. Furthermore, the majority of the participants knew about the national AMR strategic plan, although the distribution of the KAP score was significantly different across the cities (*p* = 0.046) (Table 2).

### 2.4. Participants’ Practices Regarding the Dispensing of Veterinary Antimicrobials

The majority of the participants 98.5% (67/68) provided information on the use of veterinary drugs, and about 82.4% (56/68) informed the clients about the withdrawal period. Participants in Lilongwe had higher KAP scores for the practice of providing information for the use of veterinary drugs than participants in the Blantyre and Mzuzu cities (*p* = 0.028). However, only 10.3% (7/68%) of participants claimed that they asked for written prescriptions from the clients, and very few participants, about 7.3% (5/68), were guided by written prescriptions. About half of the participants 52.9% (36/68) had an interest in knowing whether the person administering the drugs was competent (Table 3).

### 2.5. Participants’ Attitude Regarding Dispensing of Antimicrobials

The study found that 70.6% (48/68) of participants considered that the occurrence of AMR in livestock was severe, and 89.7% (61/68) of the participants considered drug residue was responsible for AMR in livestock and humans. In addition, 67.7% (46/68) considered careless use of drugs contributed to AMR in livestock, although the distribution of KAP scores was significantly different among the cities—16 for Blantyre, 22 for Lilongwe, and 8 for Mzuzu (*p* = 0.021). A higher proportion of participants 83.8% (57/68) considered that antibiotics should be prescribed only by veterinarians and the distribution of scores was different among the cities (*p* = 0.011) (Table 4).

### 2.6. Mean KAP Scores across the Sociodemographic Characteristics

The study found that male respondents had higher mean KAP scores of 30.6 ± 7.4, 28.7 ± 13.7, and 32.3 ± 4.7 than females for knowledge, attitude, and practices, *p*-0.022, *p*-0.015, and *p*-0.039, respectively. Mean scores for the 18–35 age group were higher 29.6 ± 8.1, 27.2 ± 14.3, and 31.3 ± 5.2 than the ≥35 age group for knowledge, attitude, and practices, *p*-0.017, *p*-0.022, and *p*-0.024, respectively (Table 5). Furthermore, the mean KAP scores were significantly different among the cities and the location of veterinary shops. The overall mean KAP scores were 46.7 ± 9.6, 49.2 ± 4.7, and 41.6 ± 19.0 for knowledge, attitude, and practice, respectively, and the ranges of scores were 33–58, 7–67, and 46–67 for knowledge, attitude, and practice, respectively. The mean score for veterinary surgeons was constant at 3.0 ± 0.0 for knowledge, attitude, and practice, unlike the nonparavets and paravets (Table 5).

### 2.7. Distribution of Participants‘ KAP Scores across the Categories

The scores obtained by participants across the three categories were similar for knowledge and practices (47.1%, 32/68) but different for attitude (55.9%, 38/68), (Table 6).

### 2.8. Analysis of the Association between AMR High-Level Participants and the Potential Determinants

Over half of the participants, 52.9% (36/68) had a high-level KAP score for AMR at a cutoff point of 50% and above. Pearson chi-square was run to assess the association between the potential determinants and AMR knowledge. There was an association between high level and the potential determinants such as age (*p* = 0.015); sex (*p* = 0.001), location of shop (*p* = 0.001), work experience (*p* = 0.001), knowledge of the occurrence of AMR in livestock and humans (*p* = 0.001), the practice of asking for a written prescription (*p* = 0.001), and the practice of informing customers on withdrawal period (*p* = 0.001). Thereafter, the variables were screened for multicollinearity using univariate linear regression (Table 7).

### 2.9. Determinants of AMR KAP Level for the Participants

The significant determinants of AMR KAP were sex, age, and the practice of asking for a written prescription. Odds ratios (OR) are presented in Table 8. Participants that asked for a written prescription were OR: 16.291 (95% CI: 11.6–24.2) times more likely to be knowledgeable about AMR than participants that were not asking for a written prescription (*p* = 0.024). Female participants were OR: 0.609 (95% CI: 0.3–0.9) times less likely to be knowledgeable about AMR than male participants that knew AMR (*p* = 0.001), and older participants (≥35) were OR: 0.227 (95% CI: 0.1–0.5) times less likely to be knowledgeable about AMR than participants that knew AMR (*p* = 0.004).

## 3. Discussion

This is the first report on the assessment of KAP of veterinary drug dispensers regarding AMR in Malawi. The results of this study demonstrated that there was poor knowledge, negative attitudes, and poor practices among most of the veterinary drug dispensers toward AMR in the three cities that were sampled. The most dispensed class of antibiotics was tetracycline. The following were the observed determinants: the practice of asking for a written prescription, female, and old age of the veterinary drug dispensers.

The current study’s finding that the overall mean KAP score for knowledge, attitude, and practice is suboptimal is similar to results in Nigeria, Myanmar, and Morocco [28,29,30]. The study observed differences in some of the sociodemographic aspects such as sex, education level, and age (Table 5). Male participants had higher mean scores possibly because they were principal proprietors with qualifications in animal health. As such, their level of knowledge of issues about AMR was better than female participants. The differences in mean scores between the young age and old age group categories could be attributed to the observation that the majority of paravets were recent young graduates and their curriculum included AMR-related topics. This also could influence the mean score for the young age as previously reported [31,32,33]. The mean score results for sex and age were in tandem with the multivariate results, which suggested that females and older participants were less likely to be knowledgeable about AMR, a finding which is in agreement with a report from Ethiopia [34]. Another difference in mean score was observed among the cities, between the location of shops, and types of shops. Lilongwe had a better score, possibly because as a capital city, there are more interactions with both knowledgeable natives and foreign farmers who take special care and responsibility toward observing AMR control measures [32]. In addition, although this was not investigated in this study, we speculate that Lilongwe being more affluent than Blantyre and Mzuzu, the people in the capital city may have more access to AMR information through social media networks than those in other cities. The same reasoning could be applied to the observed differences in mean scores for the participants from town centers as opposed to shops located in residential areas. The difference in mean score for the type of shops would be due to the wide difference in participants between the poultry-specific shops and general veterinary drugs shops. Many participants practiced general veterinary drugs shops compared to the poultry-specific shops due to business diversifications (Table 1). However, the general vet shops had a wide range of brands for antimicrobials, which probably resulted in compromised knowledge of each drug in stock, and hence delivery of poor quality veterinary drug services, including antimicrobial stewardship.

In addition, the study observed that aliseryl, a brand of antibiotics composed of tetracycline, erythromycin, streptomycin, colistin, and vitamins, is used for noneffective dose as a growth promoter, feed efficiency booster, and production booster on a daily basis in livestock as previously reported in more than seven countries [17,35]. This suggested that humans take antibiotics when consuming foods of animal origin obtained from such livestock products as previously reported [35,36,37] and possibly cause health risks in humans, including AMR [35]. The mean score for veterinary surgeons was lowest at 3.0 ± 0.0 in all three categories of KAP because the group had few members/participants (3), unlike paravets and nonparavets. Despite that, they performed excellently in all sections. A similar result was reported in Nigeria [38], but the results contradicted the report [39]. This could be a result of the complete veterinary background. This suggested that veterinary surgeons might be well positioned to manage the role of veterinary drug dispensers because veterinarians had more knowledge of AMR due to their training, followed by paravets, then nonparavets [38,40]. Therefore, this should be a point for a thorough assessment for better implementation of AMR control and prevention in Malawi [41].

Furthermore, our findings suggested insufficient knowledge about antimicrobial usage, residue, and resistance among veterinary drug dispensers similar to what was reported in Morocco [30]. Moreover, there were misunderstandings due to a lack of knowledge about antimicrobial use (AMU), with respondents believing that antibiotics can cure all diseases and have similar curative effects. We found this knowledge strange and may drive the careless and/or indiscriminate use of antimicrobial drugs, especially antibiotics [42]. This finding agreed with the belief by half of the participants that antibiotics should be administered to all animals when one animal is sick as previously reported in Pakistan [43]. Most participants considered that antibiotics are not the best option for viral infections and were aware of the national AMR strategic plan, which suggested that there is potential for adherence to guidelines given proper awareness and enforcement of regulations suggested [44,45]. Despite knowing the occurrence of AMR in livestock and humans, the participants had poor overall KAP scores for knowledge of AMR. We believe that the main source of AMR in livestock is a veterinary drug dispenser, which could also be a critical control point to contain the growing challenge in the country [46]. Therefore, achieving the practice of judicious AMU among animal and pet owners will be dependent on customized training on veterinary drug dispensers that factor in stewardship.

Like pharmacists in the medical field, veterinary drug dispensers have a role in controlling antibiotic abuse and the spread of AMR [41,46]. In our study, more than half of the veterinary drug dispensers (52.9%) had the interest to know the person to administer the medicine (Table 3). Unfortunately, small proportions (10.3%) of participants asked for a written prescription from the clients, and few participants about (7.3%) were guided by written prescriptions similar to the report [47]. This could be driven by a business mindset among veterinary drug dispensers as well as the desire to dispose of the medicine before its expiry date [48]. This was evident with the practice of selling products that were about to or expired at a low price to avoid losses. In addition, this was consistent with the findings of regression that participants who did not ask for a written prescription were less likely to know about AMR. The study found that almost every participant (98.5%) provided information on how to use the medicine probably as part of customer care service to please the customer, build confidence, and maintain the relationship.

The participants’ attitude was suboptimal, despite obtaining good scores on some questions. Participants’ perception toward specific areas was encouraging and could be used as a cornerstone for the improvement of AMR stewardships [49]. Most participants considered the use of antibiotics as growth promoters as a bad practice and attributed drug residues to the occurrence of AMR in livestock and humans. This consciousness and perception could be driven by brotherly love and remorse for not doing the right thing for the love of business. Worth noting was that majority of participants considered that veterinarians should be providing prescriptions which were evident through the observed association in bivariate analysis (Table 7). This was consistent with results that participants that had a practice of asking for a written prescription were more likely to be knowledgeable of AMR than participants that had no experience with this practice (Table 8). This practice was most likely driven by an attitude of the current severity of AMR both in livestock and humans. If the observed attitude could be coupled with proper action, we could progress in the right direction concerning antimicrobial stewardship and achieve a lot in the fight against AMR in Malawi. However, the only limitation of the study was the failure to present attitude questions in Likert scale format since it required more time for administration, hence distracting time for business as observed during the pilot of the questionnaire.

## 4. Materials and Methods

### 4.1. Description of the Study Sites

**Figure 2:**
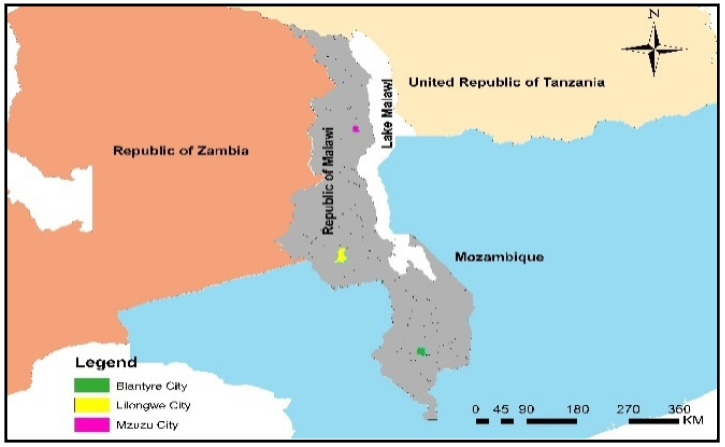
The locations of the three cities.

The study was conducted in three main cities of Malawi, namely, Blantyre, Lilongwe, and Mzuzu (Figure 2). These three cities have most of the agrovet shops in the country. Mzuzu city is the central source of veterinary products/drugs to six districts found in the northern region of Malawi, while Lilongwe city caters for the districts in the central region. Lilongwe also serves some districts that belong to the southern region such as Machinga, Mangochi, Zomba, and Balaka districts. Blantyre city is the first option for farmers in the southern region. It serves 13 districts, including the major dairy production areas under Shire Highland Milk Production Association (SHMPA).

### 4.2. Study Design, and Sample Size

A cross-section study was conducted in three main cities of Malawi, namely, Blantyre, Lilongwe, and Mzuzu because of the presence of main agrovet shops (Figure 2). The cities were purposively selected, while a simple random sampling technique was used to select veterinary drug dispensers in these cities from a population size of 137. The sampling unit was the agrovet shop; either the shop owner or shop attendant was interviewed. An agrovet shop was defined as a shop that had a certificate, from the Pharmacy and Medicine Regulatory Authority (PMRA), to practice the trade of veterinary products. These veterinary products included feeds, feed ingredients, and drugs for livestock use. The participants were randomly selected from a list provided by PMRA Registrar. In the absence of the owner, the agrovet shop is managed by a shop attendant who may not have a veterinary background. Attendants without veterinary background are defined as nonparavets, whereas those having certificates or diplomas in animal health are defined as paravets. The study was carried out from July to November 2022. The Raosoft online sample size calculation [49] was used to determine the number of participants based on the following conditions: 5% margin of error (population size was 137), 85% confidence level, and assumption of response distribution of 50% [49], after adding a 5% nonresponse rate. The minimum sample size was 83, and it was proportionally distributed as 15 Mzuzu, 40 Lilongwe, and 28 Blantyre.

### 4.3. Data Collection Instruments

A structured questionnaire was developed principally based on questionnaires used in previous similar studies [50,51,52,53]. The survey questionnaire was partitioned into four major sections. Firstly, the questionnaire obtained sociodemographic information such as age, sex, education, type of veterinary drug shop, location of the shop, and specialty. The second section obtained information on knowledge of antimicrobials, AMU, AMR, and antimicrobial residue. The participants’ attitudes and practices regarding AMU, AMR, and antimicrobial residues were assessed in the third and fourth sections, respectively.

### 4.4. Questionnaire Validation and Data Collection

Questionnaires were prevalidated for relevance, accuracy, clarity, simplicity, and understandability and Cronbach’s alpha coefficient of 0.74, 0.76, and 0.72 for knowledge, practice, and attitude, respectively, indicating the internal consistency and reliability of the study questionnaires [54]. The questionnaires were administered on tablets using Epicollect5 software and the pilot study included 10% of the sample size that was excluded from the final analysis. Additionally, sufficient training was provided to enumerators to clear the observed discrepancies and improve the quality of the collected information. The training was conducted at the Department of Epidemiology and Public Health, Faculty of Veterinary Medicine, Lilongwe University of Agriculture and Natural Resources (LUANAR). Data were collected through face-to-face interviews following verbal consent from participants.

### 4.5. Data Interpretation, Processing, and Analysis

Data for the five most antibiotics on demand were grouped according to the class of active ingredients. The frequency of the antibiotic classes was calculated based on the number of responses.

Scores for knowledge, attitude, and practice were calculated by adding correct/positive responses (coded as 1 for correct/positive response, and otherwise zero) as previously reported by [26,55]. Good knowledge was determined based on overall scores above 80%; moderate knowledge was scored from 50% to 80% and low knowledge was scored below 50%. A similar assessment scale was used for practices, good practice was above 80%, the moderate practice was scored from 50% to 80%, and poor practice was scored below 50%. Positive attitudes were scores of 63% and above, while negative attitudes were scores below 63% [26,56]. The high-level participants were obtained by the total sum of each participant’s scores, and a cutoff of 50% was used [56]; then, comparisons were conducted using Student’s *t*-test and the chi-square test [57].

The response alternatives for knowledge, practice, and attitude were mostly dichotomous, presented as “yes” or “no”, meaning “true” or “false”, respectively. Data were cleaned and validated in Microsoft™ Excel Spreadsheet (Microsoft Office Excel^®^2019. Data analysis was conducted using SPSS Ver. 21 (IBM Corp, Armonk, NY, USA) statistical software. Before data analysis, every correct response was accorded a value of 1 and otherwise, and the response was accorded a 0 value. The normality test was done graphically using QQ-plots and Shapiro–Wilk test; thereafter, the Student *t*-test or One-Way ANOVA was used to compare the mean differences across explanatory variables for knowledge, attitude, and practice scores. The Chi-square test was used to assess the association between AMR knowledge, attitude, and practice and independent variables. Multicollinearity was checked using Variance Inflation Factors (VIF) (VIF value < 1.00) and Tolerance (value > 0.20). Thereafter, a multivariable linear regression model was fitted, which included variables that retained significance (*p* < 0.05) at univariate analysis [26,55]. The generated multivariable model was tested for goodness of fit and predictability using the Hosmer–Lemeshow test and Omnibus test, respectively. Stepwise regression method and Enter algorithms were used, and independent variables with a *p*-value less than 0.05 were considered significant predictors of AMR knowledge, attitude, and practice.

## 5. Conclusions

This study found poor knowledge, negative attitudes, and poor practices concerning AMR among most of the participants. Sensitization and antimicrobial stewardship training are recommended to address the KAP score gaps and the observed determinants among veterinary drug dispensers. The government should consider ways to scale up its regulations on veterinary drug dispensers. It is imperative to conduct a similar future study among veterinarians to ascertain the reported results. Subsequently, it is critical to strengthen antimicrobial stewardship programmers in the veterinary sector in Malawi.

## Figures and Tables

**Table 1:** Sociodemographic characteristics of study participants.

Sociodemographic Characteristics	Frequency	Proportion	95% CI
	(*n* = 68)	(%)	
Sex			
Female	21	30.9	20.5–43.4
Male	47	69.1	56.6–79.5
Age (years)			
18–35	47	69.1	56.6–79.5
≥35	21	30.9	20.5 43.4
Specialty			
Non-paravet	14	20.6	12.1–32.5
Paravet	51	75	62.8–84.4
Vet surgeon	3	4.4	1.1–13.2
Level of education			
Primary school and below	3	4.4	1.1–13.2
Secondary school	11	16.2	8.7–27.5
Tertiary	54	79.4	67.5–87.9
Work experience (years)			
<1	11	16.2	8.7–27.5
1–5	32	47	34.9–59.5
≥5	25	36.8	25.6–49.4
Type of vet shop			
Poultry-only vet products shop	4	5.9	1.9–15.1
General agrovet shops	64	94.1	84.9–98.1
Location			
Within town center	56	82.4	70.8–90.2
Within residential area	12	17.6	9.8–29.2
City			
Mzuzu	12	17.7	9.8–29.2
Lilongwe	32	47	34.9–59.5
Blantyre	24	35.1	24.4–47.9

*n* = number of participants.

**Table 2:** The proportion of respondents who correctly/positively responded to knowledge questions on antimicrobial use and resistance across the study area.

Knowledge Questions	Total *n* = 68 (%)	Blantyre *n* = 24	Lilongwe *n* = 32	Mzuzu *n =* 12	*p*-Value
Knowledge of uses of antibiotics (Yes)	51 (75.0)	20 (83.3)	22 (68.8)	9 (75.0)	0.051
Antibiotics can cure all diseases caused by microorganisms in livestock (No)	33 (48.5)	10 (41.7)	12 (37.5)	11 (91.7)	0.664
All antimicrobials have the same curative effect in livestock diseases, true or false (False)	56 (82.4)	19 (79.2)	29 (90.6)	8 (66.7)	**0.037**
Knowledge of the antimicrobials having similar adverse effects in livestock and humans (No)	35 (51.5)	13 (54.2)	12 (37.5)	10 (83.3)	0.276
Knowledge of AMR (Yes)	52 (76.5)	19 (79.2)	25 (78.1)	8 (66.7)	0.921
Knowledge of the occurrence of AMR in livestock and humans (Yes)	52 (76.5)	19 (79.2)	25 (78.1)	8 (66.7)	0.921
Are antimicrobials required for all livestock when one animal is sick (No)	35 (51.5)	13 (54.2)	12 (37.5)	10 (83.3)	0.276
Knowledge of products that have antibiotics as growth promoters (Yes)	51 (75.0)	18 (75.0)	25 (78.1)	8 (66.7)	0.064
Knowledge of the National AMR strategic plan	55 (80.9)	20 (83.3)	28 (87.5)	8 (66.7)	**0.046**
Antibiotics are not the best option for the control of viral infections, is it true (Yes)	51 (75.0)	17 (70.8)	25 (78.1)	9 (75.0)	0.182

*n* = number of respondents; % = Percentage; boldface indicates statistical significance at *p* < 0.05.

**Table 3:** The proportion of respondents who correctly/positively responded to practice questions regarding dispensing of antimicrobials across the study area.

Practice Questions	Total *n* = 68 (%)	Blantyre *n* = 24	Lilongwe *n* = 32	Mzuzu *n =* 12	*p*-Value
Do you ask for a written prescription from your clients before issuing the drugs? (Yes)	7 (10.3)	3 (12.5)	2 (6.2)	2 (16.7)	0.064
What guides your selection of antibiotics to sell? (written prescription)	5 (7.3)	3 (12.5)	1 (3.1)	1 (8.3)	0.059
Do you provide information on how to use the dispensed drugs? (Yes)	67 (98.5)	24 (100.0)	31 (96.9)	12 (100.0)	**0.028**
Do you inform customers of the withdrawal period? (Yes)	56 (82.4)	19 (79.2)	29 (90.6)	8 (66.7)	**0.038**
Do you observe expiry dates for the antibacterials available? (Yes)	61 (89.7)	22 (91.7)	29 (90.6)	10 (83.3)	0.051
How do you dispose of expired drugs? Destroy them	40 (58.8)	16 (66.7)	15 (46.9)	9 (75.0)	0.087
Do you make a follow-up on drug usage with farmers that buy antibiotics here?	42 (61.8)	16 (66.7)	16 (50.0)	10 (83.3)	0.215
How do you keep your drugs? Cool dry place	61 (89.7)	22 (91.7)	29 (90.6)	10 (83.3)	0.051
Do some of your products have antibiotics as growth promoters? (Yes)	33 (48.5)	12 (50.0)	16 (50.0)	5 (41.6)	0.169
Do you follow up on reports concerning antibacterial nonresponsive infection? (Yes)	42 (61.8)	16 (66.7)	15 (46.9)	11 (91.7)	0.072
Do you find out who administers the antibiotics? (Yes)	36 (52.9)	13 (54.2)	12 (37.5)	11 (91.7)	0.051

*n* = number of respondents; % = Percentage; boldface indicates statistical significance at *p* < 0.05.

**Table 4:** The proportion of respondents who correctly/positively responded to attitude questions regarding dispensing of antimicrobials across the study area.

Attitude Questions	Total *n* = 68 (%)	Blantyre *n* = 24	Lilongwe *n* = 32	Mzuzu *n =* 12	*p*-Value
How do you feel towards antibacterial resistance occurrence in livestock, do you think it’s severe? (Yes)	48 (70.6)	18 (75.0)	22 (68.8)	8 (66.7)	0.621
Do you consider drug residual accounts for antibacterial resistance occurrence both in livestock and humans? (Yes)	61 (89.7)	21 (87.5)	26 (81.3)	11 (91.7)	0.056
Do you consider careless use of antibacterials in livestock may lead to AMR? (Yes)	46 (67.7)	16 (66.7)	22 (68.8)	8 (66.7)	**0.021**
What is your opinion on selling the antibacterials at a lower price when about to expire to prevent wastage, is it a risk to antibacterial resistance? (Yes)	49 (72.1)	19 (79.2)	22 (68.8)	8 (66.7)	0.224
How do you feel, if antibacterial residues can be passed to humans from livestock products, is it dangerous to human health? (Yes)	49 (72.1)	19 (79.2)	22 (68.8)	8 (66.7)	0.224
Do you consider AMR a problem that causes many health challenges in humans? (Yes)	49 (72.1)	19 (79.2)	22 (68.8)	8 (66.7)	0.224
Do you consider low awareness of AMR among veterinary drug dispensers, to be a drawback to the prevention of drug residual and AMR in livestock and humans? (Yes)	49 (72.1)	19 (79.2)	22 (68.8)	8 (66.7)	0.224
Do you consider the use of antibacterials may be reduced by maintaining proper biosecurity, vaccination, and good management practices? (Yes)	49 (72.1)	19 (79.2)	22 (68.8)	8 (66.7)	0.224
Do you consider that antibiotics should be prescribed only by veterinarians? (Yes)	57 (83.8)	21 (87.5)	27 (84.4)	9 (75.0)	**0.011**
Do you consider it bad towards using antibiotics as growth promoters in livestock production? (Yes)	55 (80.9)	20 (83.3)	26 (81.3)	9 (75.0)	0.126

*n* = number of respondents; % = Percentage; boldface indicates statistical significance at *p* < 0.05.

**Table 5:** Mean knowledge, attitude, and practice scores across sociodemographic characteristics.

Variable	Level	Knowledge		Practice		Attitude	
		Mean	STD. Deviation	Mean	STD. Deviation	Mean	STD. Deviation
Sex	Male	30.6	7.4	28.7	13.7	32.3	4.7
	Female	16.0	2.2	12.8	5.4	18.9	0.3
*p*-value		**0.022**		**0.015**		**0.039**	
Age (years)	18–35	29.6	8.1	27.2	14.3	31.3	5.1
	≥35	17.1	1.7	14.5	5.3	19.9	0.7
*p*-value		**0.017**		**0.022**		**0.024**	
Education Level	Primary and below	0.9	0.6	0.7	0.6	1.0	0.7
	Secondary leaver	5.9	1.8	4.7	1.6	3.7	1.3
	Tertiary	38.9	8.4	36.4	17.3	46.4	4.1
*p*-value		**0.044**		**0.049**		0.078	
Specialty	Nonparavet	3.2	1.6	3.0	3.6	8.9	2.3
	Paravet	40.4	7.9	35.5	16.4	38.8	6.9
	Vet surgeon	3.0	0.0	3.0	0.0	3.0	0.0
*p*-value		0.051		0.069		**0.041**	
Work experience (years)	<1	5.3	1.7	4.9	3.9	4.9	2.7
	1–5	23.6	4.0	20.4	9.7	21.5	4.0
	≥5	17.7	3.9	16.1	5.8	24.8	2.1
*p*-value		**0.024**		**0.035**		0.084	
Type of vet shop	Poultry only	3.2	0.4	3.1	0.7	3.3	0.5
	General vet shop	43.4	9.4	37.7	20.4	47.9	4.8
*p*-value		**0.001**		**0.001**		**0.001**	
Location	Town centre	39.2	8.6	35.7	16.8	41.2	5.1
	Residential area	7.4	0.9	5.5	2.9	10.0	1.3
*p*-value		**0.027**		**0.036**		**0.024**	
City	Mzuzu	4.9	1.9	6.3	3.4	7.5	2.4
	Lilongwe	24.5	3.9	21.4	8.9	25.2	3.6
	Blantyre	17.4	5.8	14.3	7.5	18.5	1.7
*p*-value		**0.042**		**0.038**		**0.033**	
Overall		46.7	9.6	41.6	19.0	49.2	4.8
Range		33–58		7–67		46–67	

Std. Deviation = Standard Deviation, boldface indicates statistical significance at *p* < 0.05.

**Table 6:** Summary of grades for the participants’ KAP levels toward AMR.

Grades for Knowledge	Scale	Proportion (*n* = 68)	Grades for Practice	Scale	Proportion (*n* = 68)	Grades for Attitude	Scale	Proportion (*n* = 68)
Poor	<50	32 (47.1)	Poor	<50	32 (47.1)	Negative	<63	30 (44.1)
Moderate	50–80	29 (42.6)	Moderate	50–80	29 (42.6)	Positive	>63	38 (55.9)
Good	>80	7 (10.3)	Good	>80	7 (10.3)			
*p*-value		0.668			0.668			0.137

*n* = number of participants.

**Table 7:** Summary of bivariate analysis between potential determinants of knowledge, attitude, and practices toward AMR.

	Number Tested (*n* = 68)	High Level (*n* = 36)	Positivity (%)	OR	95% CI	*p* = Value
**Potential determinants under** **Sociodemographic Characteristics**						
Sex (*n* = 68)						
Male	47	33	70.2	ref		
Female	21	3	14.3	0.57	0.1–0.7	0.032 ***
Age (*n* = 68)						
18–35	47	29	61.7	ref		
≥35	21	7	33.3	0.011	0.0–0.8	0.001 ***
Work experience (*n* = 68)						
<1	11	6	54.6	ref		
1–5	32	17	53.1	0.414	0.0–0.9	0.053 *
≥5	25	13	52.0	0.201	0.1–0.8	0.041 ***
Location of the shop (*n* = 68)						
Town center	56	26	46.4	ref		
Residential areas	12	10	83.3	3.661	1.6–18.7	0.001 ***
**Potential determinants of knowledge category**						
All antibiotics have the same curative effect (*n* = 68)						
Yes	56	27	48.2	ref		
No	12	9	75.0	6.833	4.9–31.7	0.019 ***
Knowledge of products that have antibiotics as growth promoters (*n* = 68)						
Yes	51	31	60.8	ref		
No	17	5	29.4	0.524	0.2–0.8	0.212 *
Knowledge of the occurrence of AMR in livestock and humans (*n* = 68)						
Yes	52	31	59.6	ref		
No	16	5	31.1	0.524	0.2–0.8	0.212 *
**Potential determinants of attitude category**						
Considering bad toward using antibiotics as growth promoters in livestock production (*n* = 68)						
Yes	55	25	45.5	ref		
No	13	11	84.6	4.442	1.5–22.7	0.001 ***
Consider that antibiotics should be prescribed only by veterinarians (*n* = 68)						
Yes	57	28	35.9	ref		
No	11	8	72.7	6.77	2.9–17.1	0.021 ***
**Potential determinants under practice category**						
Do you ask for a written prescription (*n* = 68)						
Yes	15	13	86.7	ref		
No	53	23	43.4	0.010	0.0–0.7	0.003 ***
Do you inform customers of the withdrawal period (*n* = 68)						
Yes	56	29	51.8	ref		
No	12	7	58.3	6.833	4.9–31.7	0.019 ***

*n* = Number of participants; CI = Confidence interval, Significant level < 0.05; OR = Odds ratio; *** = Significant at 0.05, considered for multivariate analysis; * = considered for multivariate analysis (cut-off *p* = ≤ 0.250); ref = reference.

**Table 8:** Summary of maximum-likelihood estimates for determinants associated with AMR knowledge.

Variable	Level	OR	95% CI	*p*-Value
Sex (*n* = 68)	Men	ref		
	Female	0.609	0.3–0.9	0.001 ***
Age (*n* = 68)	18–35	ref		
	≥35	0.227	0.1–0.5	0.004 ***
Ask for a written prescription (*n* = 68)	No	ref		
	Yes	16.291	11.6–24.2	0.024 ***

*** = Significant at 0.05; OR = Odds ratio; CI = Confidence interval; Significant at *p* < 0.05; ref = Reference category.

## Data Availability

The data supporting the reported results can be made available on request from the corresponding author.

## Supplementary Materials

The following supporting information can be downloaded at: https://www.mdpi.com/article/10.3390/antibiotics12010149/s1, File S1: Study questionnaire.
