# Supplementary material for: Determinants of Knowledge, Attitude, and Practices of Veterinary Drug Dispensers toward Antimicrobial Use and Resistance in Main Cities of Malawi: A Concern on Antibiotic Stewardship
Source: Antibiotics (Basel). 2023 Jan 11;12(1):149. doi: 10.3390/antibiotics12010149 (PMC9855151; doi:10.3390/antibiotics12010149)
Supplement: Supplementary file 1 [file antibiotics-12-00149-s001.zip › antibiotics-2094961-supplementary.pdf]

i. **Data collection tool**
ii. **Participants Consent Form**

i. Questionnaire on AMR for veterinary drug dispensers in Mzuzu, Lilongwe and Blantyre

Lilongwe University of Agriculture and Natural Resources (LUANAR)

Department of Epidemiology and Public Health

Faculty of Veterinary Medicine

INTRODUCTION

This is a questionnaire aimed at collecting data on Knowledge, attitude and practices of veterinary products dispensers towards Antimicrobial resistance in major cities of Malawi: a concern on antibiotic stewardship. The quality and success of this study is highly dependent on the relevance of the information that you will provide. So, you are kindly requested to give your genuine response to each item of the questionnaire. Your response will be used solely to carry out this study and will be kept confidential.

Kindly we are asking for your time to truthfully respond to the following questions

Section A: Socio-Demographics information of respondents

1) Sex: 1) Male ☐

2) Female ☐

2) Age: 1) 18-35 ☐

2) Above 35 ☐

3) Type of vet product shop 1) Poultry vet shop ☐

2) Ruminants vet shop ☐

3) General vet shop ☐

4) Education level 1) Primary school leaver and below ☐

2) Secondary school leaver ☐

3) Tertiary education ☐

5) Specialty 1) Paravet ☐

2) Non-Paravet ☐

3) Vet surgeon

6) Work experience 1) Less than 1 year ☐

	2) 1-5 years	☐
	3) Above 5 years	☐
7) Location of shop	1) Within central markets	☐
	2) Residential areas	☐

Section B: Knowledge of respondents towards AMR

1) Do you know uses of antibiotics?
   1) Yes ☐
   2) No ☐

2) Do you know antibiotics can cure all diseases caused by microorganisms in livestock?
   1) Yes ☐
   2) No ☐

3) All antimicrobials can have the same adverse effect in human and livestock, true or false?
   True ☐
   False ☐

4) Do you know Antimicrobial Resistance (AMR) Yes ☐
   No ☐

5) Do you know antimicrobial resistance occur in both humans and livestock
   Yes ☐
   No ☐

6) Do you think antimicrobial are required to all livestock when one animal is sick?
   Yes ☐
   No ☐

7) Do your veterinary products have antibiotics as growth promoters?
   Yes ☐
   No ☐

8) Do you remind your clients on withdraw period Yes ☐
   No ☐

9) Are you conversant with National AMR strategic plan?
   Yes ☐
   No ☐

10) Antibiotics are not best option for control of viral infections in livestock
   Yes ☐
   No ☐

Section C: Practices of respondents towards dispersion of Antimicrobial drugs

1) What type of antibiotics do you have? Provide the list of most common top five antibiotics on demand

2) Do you ask for written prescription from your clients before issuing the drugs
   1) Yes ☐
   2) No ☐

3) What guides your selection of antibiotics to sell?
   1) Prescription ☐
   2) Customer preference ☐
   3) Your experience ☐

4) Do you provide information on how to use the dispersed drugs?
   1) Yes ☐
   2) No ☐

5) Do you inform customers on withdraw period?
   1) Yes ☐
   2) No ☐

6) Do you observe expiry dates for the antimicrobial drugs available?
   1) Yes ☐
   2) No ☐

7) Where do you keep antibiotics?
   1) Cool dry place ☐
   2) Wet cool place ☐

8) How do you dispose the expired drugs
   1) Sell at reduced price ☐
   2). Give away to needy farmers ☐
   3). Destroy the products ☐

9) Do some of your products have antibiotics as growth promoters
   1) Yes ☐
   2) No ☐

10) Do you follow up on reports concerning antimicrobial nonresponsive infection
   1) Yes ☐
   2) No ☐

11) Do you find out who administer the antibiotics

1) Yes ☐

2) No ☐

Section D: Attitude of respondents towards AMR

1) How do you feel towards antimicrobial resistance occurrence in livestock, do you think its severe?
   Yes ☐
   No ☐

2) Do you consider drug residual accounts for antibacterial resistance occurrence both in livestock and humans??
   1) Yes ☐

   2) No ☐

3) Do you consider careless use of antibacterial drugs in livestock may lead to AMR?
   1) Yes ☐

   2) No ☐

4) What is your opinion on selling the antibacterials at a lower price when about to expire to prevent wastage, is it risk to antibacterial resistance?
   Yes ☐
   No ☐

5) How do you feel, if antibacterial residues can be passed to humans from livestock products, is it dangerous to human health?
   1) Yes ☐

   2) No ☐

6) Do you consider AMR as a problem that causes many health challenges in humans?
   1) Yes ☐

   2) No ☐

7) Do you consider low awareness of AMR among veterinary drug dispensers, is a drawback to prevention of drug residual and AMR in livestock and humans
   1) Yes ☐

   2) No ☐

8) Do you consider the use of antibacterial may be reduced by maintaining proper biosecurity, vaccination, and good management practices
   1) Yes ☐

   2) No ☐

9) Do you consider that antibiotics should be prescribed only by veterinarians?

1) Yes ☐
2) No ☐

10) Do you feel bad towards using antibiotics as growth promoters in livestock production?

1) Yes ☐
2) No ☐

Thank you for your cooperation!

ii. **Participants` information sheet and consent form**

Title of Study: Determinants of Knowledge, Attitude, and Practices of Veterinary Drug Dispensers towards Antimicrobial Use, Resistance and Residue in Main Cities of Malawi: A Concern on Antibiotic Stewardship

Principal Investigator: Dr. Henson Kainga
Co-Principal Investigator: Dr. Mike Luwe

The study aimed at collecting data on Knowledge, attitude and practices of veterinary products dispensers towards Antimicrobial resistance in major cities of Malawi: a concern on antibiotic stewardship. The quality and success of this study is highly dependent on the relevance of the information that you will provide. So, you are kindly requested to give your genuine response to each item of the questionnaire. Your response will be used solely to carry out this study and will be kept confidential. As we discuss the information below, please feel free to ask any questions.

If you do not join the study, you will not be victimised in any way. You may choose to leave the study at any time you like. This exercise will take about 20-30 minutes. The questions that will be asked are general and not personal. There should be no risks to you if you agree to take part in the study.

You will not directly benefit from this study. The information we collect will be used to improve antibiotic use and antibiotic stewardship in the fight against Antimicrobial Resistance (AMR). ***THERE IS NO MONEY TO BE PAID TO YOU BY PARTICIPATION***.
The information that will be collected in this study will be confidential and will be used in academic manner only and not necessarily linked to particular individuals.

If you have questions, complaints, or problems as a result of participating in this study, you may call Henson Kainga (+265 882 675 428 / 0996 618 212 or email: kaingahenson@yahoo.com. You can also consult the Director of Veterinary Research and Investigation (Dr. Julius Chulu) on +265 991277492, Department of Animal Health and Livestock Development in Ministry of Agriculture and Water Development, Republic of Malawi. You can further verify our study by contacting Mr. Edwin Chipala of Pharmacy and Medicine Regulatory Authority (PMRA), P.O. Box: 30241, Lilongwe; echipala@mpra.mw or call 0997315964 / 0212755165

What does your signature (or thumbprint/mark) on this consent form mean?

Your signature **(or thumbprint/mark)** on this form means:

- You have been informed about this study's purpose, procedures, possible benefits and risks.
- You have been given the chance to ask questions before you sign.
- You have voluntarily agreed to participate in this study.

Please indicate Yes or No

- I agree to allow my livestock to participate in this study ☐Yes ☐No
- I agree to be interviewed and allow data from my questionnaire to be used in the study ☐Yes ☐No

Name of participant: ____

______________________________ ________________

Signature of participant or Thumb print Date

In signing here, I agree that I have read and understood the agreement/consent form and agree to participate in the study.

Name of Witness: ____

_________________________________ ________________

Signature of Witness Date

______________________ ________________
Signature of Enumerator Date
